# Prevalence of allergic rhinitis, atopic dermatitis, and wheezing at 15 and 22 years of age: the 1993 Pelotas (Brazil) Birth Cohort Study

**DOI:** 10.36416/1806-3756/e20240317

**Published:** 2024-11-26

**Authors:** Gabriela Ávila Marques, André F. S. Amaral, Valéria Lima Passos, Priscila Weber, Paula Duarte de Oliveira, Ana Maria Baptista Menezes, Helen Gonçalves, Fernando César Wehrmeister

**Affiliations:** 1. Programa de Pós-Graduação em Epidemiologia, Universidade Federal de Pelotas, Pelotas (RS), Brasil.; 2. Instituto Nacional do Coração e Pulmão, Imperial College London, Reino Unido.; 3. Escola de Farmácia e Ciências Biomoleculares, Colégio Real de Cirurgiões da Irlanda, Irlanda.; 4. Empresa Brasileira de Serviços Hospitalares - EBSERH, Brasília (DF), Brasil.; 5. Instituto de Saúde Pública Global, Universidade de Manitoba, Canadá.

**Keywords:** asthma, wheezing, allergic rhinitis, atopic dermatitis, epidemiology

## Abstract

**Objectives:**

To estimate the prevalence of allergic rhinitis (AR), atopic dermatitis (AD), and wheezing, and to describe their patterns of co-occurrence according to different characteristics in adolescence and early adulthood.

**Methods:**

Cross-sectional analyses from the 15-year and 22-year follow-ups of the 1993 Pelotas (Brazil) Birth Cohort. The outcomes were assessed based on self-reported data, and the patterns of co-occurrence were determined using cluster analysis. The sample was described using absolute and relative frequencies according to the independent variables. Venn diagrams were generated to visualize the co-occurrence of AR, AD, and wheezing.

**Results:**

Data on AR, AD, and wheezing were available for 4,286 participants at 15 years and 3,789 at 22 years. At 15 years, AR was reported by 20.9% of participants, AD by 25.2%, and wheezing by 33.4%. Meanwhile, at 22 years, AR was reported by 24.6%, AD by 14.2%, and wheezing by 30.7%. Notably, the overlap between AR and wheezing was greater than that of the other conditions (6.9% at 15 years and 8.3% at 22 years). Participants with lower maternal education and lower income were more likely to report having “no health condition”. At 15 years, White individuals most frequently reported “three conditions” (4.1%; p<0.001), whereas at 22 years, they primarily reported “two conditions” (15.6%; p<0.001). The co-occurrence of all three health conditions was found to be greater than expected, with an observed rate 2.1 times higher (95% CI 1.4 - 3.0) at 22 years.

**Conclusions:**

This study highlights the social gradient in the diagnosis and reporting of co-occurrence of AR, AD, and wheezing.

## INTRODUCTION

Allergic asthma is more common in childhood.^1^ ^,^ ^2^ This phenotype is associated with a history of allergic rhinitis (AR), atopic dermatitis (AD), or both,^1^ ^,^ ^2^ and its symptoms are typically triggered by allergens, such as pollen and dust mites.^2^

When considered separately, the prevalence of asthma, AR, and AD varies widely within and across countries.^3^ ^-^ ^5^ In 2019, over 260 million people were diagnosed with asthma.^3^ Population-based studies have reported prevalence estimates for AR ranging from 1.0% to 63.0% in adolescents and adults globally,^4^ ^,^ ^6^ while the prevalence of AD has been shown to range from 3.4% to 33.7%.^4^ ^,^ ^5^ ^,^ ^7^

Temporal changes in the prevalence of these health conditions in childhood are described by the concept of the “atopic march”.^8^ ^,^ ^9^ This widely accepted hypothesis outlines a cascade of symptoms that typically begins with AD, which progresses to asthma and then AR, with AD often resolving as age increases.^10^ However, some researchers argue that the natural history of these conditions may vary, exhibiting different developmental symptom profiles over time.^11^ ^,^ ^12^

The co-occurrence of asthma, AR, and AD has been observed to be more common than expected.^13^ ^,^ ^14^ In the 1993 Pelotas (Brazil) Birth Cohort Study, participants with persistent symptoms of asthma, such as wheezing, had significantly higher odds of being diagnosed with an allergy (OR 6.18; 95% CI: 3.59 - 10.61) compared to those with never/infrequent wheezing.^15^ However, despite the increasing prevalence of these conditions among adolescents and young adults in lower- and upper-middle-income countries,^1^ ^,^ ^16^ ^,^ ^17^ few population-based studies have described the co-occurrence of all three conditions. 

Pedersen et al. (2020) reported that the prevalence of having two atopic conditions peaked at 10.0% at 2 years of age and was approximately 3.4% among adults in a rural area of India.^16^ In Brazil, the prevalence of these health conditions has also mostly been studied separately,^18^ ^-^ ^20^ especially among adolescents and young adults.^15^ ^,^ ^21^ ^,^ ^22^

The co-occurrence of asthma, AD, and AR reduces quality of life, rest, and academic performance among adolescents.^23^ ^-^ ^25^ Further investigation is essential to better understand these combinations and to develop more effective health interventions that mitigate their effects.^23^ ^,^ ^24^ This is particularly important during the transition from adolescence to adulthood due to its impact on long-term health outcomes and well-being.^26^ ^,^ ^27^ Therefore, the objectives of the present study were: 1) to estimate the prevalence of allergic rhinitis, atopic dermatitis, and wheezing (as a proxy for asthma), both individually and jointly; and 2) to describe the frequency of their patterns of co-occurrence according to sociodemographic, behavioral, and health characteristics in adolescence and early adulthood, based on cross-sectional analyses from the 1993 Pelotas (Brazil) Birth Cohort.

## METHODS

### Study participants

The 1993 Pelotas (Brazil) Birth Cohort is a longitudinal, prospective, and population-based study that included all live births that occurred in hospitals in the city of Pelotas (RS), Brazil, in 1993. Among the 5,265 newborns, 5,249 (99.7%) were enrolled in this cohort study. From birth to the 22-year follow-up, subsamples have provided information on the participants’ health. The present study used data collected in the 15-year and 22-year follow-ups, with response rates of 85.7% and 76.3%, respectively. The methodology, rationale, and updates of the cohort study have been described elsewhere.^28^

### Outcomes

Three health conditions were assessed at ages 15 and 22 based on self-reported data:

1. Allergic rhinitis: “Have you ever been diagnosed with allergic rhinitis?”
2. Atopic dermatitis: “Have you ever been diagnosed with eczema?”
3. Wheezing: “Have you ever had wheezing or a whistling sound in your chest any time in the past?”

### Covariates

The independent variables were measured at baseline (perinatal) and at the 15-year and 22-year follow-ups. At baseline: sex (male, female); skin color (White, Black, or Mixed); maternal age (≤ 19 years, 20-29 years, 30-39 years, ≥ 40 years); maternal education (0-4 years, 5-8 years, 9-11 years, ≥ 12 years); monthly income in multiples of minimum wage, equivalent to approximately US$ 240.00 (≤ 1, 1.1-3, 3.1-6, 6.1-10, ≥ 10); family history of allergy (no, yes); family history of asthma (no, yes); maternal smoking during pregnancy (no, yes); and birth weight (< 2500 g, ≥ 2500 g). At the 15-year and 22-year follow-ups: smoking status (never, former, or current) and weight status (normal weight, overweight, obese).

### Statistical analysis

This cross-sectional study evaluated both follow-ups independently, and only individuals with complete data for all three health conditions (AR, AD, and wheezing) were included.

The co-occurrence of AR, AD, and wheezing was determined by cluster analysis. The observed-to-expected (O/E) ratio for each combination was calculated.^29^ ^,^ ^30^ The expected prevalence for each of the eight possible combinations was determined by multiplying the observed prevalence of each health condition by the inverse of the observed proportion of missing conditions, assuming the independence of each condition. A cluster was considered statistically significant if the O/E ratio was greater than 1 and its respective 95% confidence interval (95% CI) did not include the unit (1).^29^ ^,^ ^31^ The analyses and their respective 95% CIs were stratified by sex and conducted using Microsoft Excel, version 16.85 (Microsoft Corporation, Redmond, WA, USA). 

The sample was described using absolute and relative frequencies based on the independent variables. Pearson’s chi-square test or the chi-squared test for linear trends was applied, depending on the nature of the variables. A significance level of 5% was set for all tests. In order to visualize the simultaneous occurrence of AD, AR, and wheezing, Venn diagrams were generated, with each circle representing one of the three health conditions. These analyses were conducted using Stata software, version 18.0 (StataCorp LP, College Station, TX, USA).

### Ethical aspects

Ethical approval for all follow-up visits was obtained from the Ethics and Research Committee of the Faculty of Medicine at the Federal University of Pelotas (UFPel). Informed consent was secured from all cohort members or from their guardians when the participants were under 18 years of age.

## RESULTS

Data for all three health conditions were available for 4,286 participants at the 15-year follow-up and for 3,789 participants at the 22-year follow-up. Compared to those not included in the analysis, individuals with complete data were more likely to be female and less likely to have low maternal education (0-4 years) and low birth weight at both follow-ups (Supplementary Table S1).

The characteristics of the participants at each time point are shown in Table 1. At both follow-ups, most participants were female (51.1% and 53.2%), White (66.5% and 65.8%), and had mothers who had studied less than nine years (75.1% and 73.5%). At 15 years of age, 14.5% reported a family history of allergy, while 14.6% reported a family history of asthma. At 22 years of age, both were 19.5%. Similar proportions of participants had mothers who smoked during pregnancy (33.2% and 32.7%) at both follow-ups. 

**Table 1:** Characteristics of the participants who completed the questionnaires at the 15- and 22-year follow-ups: the 1993 Pelotas Birth Cohort, Brazil.

	At the 15-year follow-up (n = 4,286)		At the 22-year follow-up (n = 3,789)	
	N	(%)	N	(%)
Sex				
Female	2,192	(51.1)	2,015	(53.2)
Male	2,094	(48.9)	1,774	(46.8)
Skin color				
Black/Mixed	1,383	(33.5)	1,171	(34.2)
White	2,744	(66.5)	2,251	(65.8)
Maternal age (years)				
≤ 19	736	(17.2)	658	(17.4)
20 - 29	2,270	(53.0)	2,016	(53.2)
30 - 39	1,187	(27.7)	1,031	(27.2)
≥ 40	92	(2.2)	84	(2.2)
Maternal education (years)				
0 - 4	1,167	(27.3)	1,007	(26.6)
5 - 8	2,047	(47.8)	1,774	(46.9)
9 - 11	736	(17.2)	698	(18.4)
≥12	329	(7.7)	305	(8.1)
Income (minimum wage)				
≤ 1	769	(18.3)	660	(17.7)
1.1 - 3	1,777	(42.2)	1,542	(41.5)
3.1 - 6	1,017	(24.2)	924	(24.8)
6.1 - 10	333	(7.9)	312	(8.4)
>10	310	(7.4)	281	(7.6)
Family history of allergy				
No	3,665	(85.5)	3,236	(85.4)
Yes	621	(14.5)	553	(14.6)
Family history of asthma				
No	3,450	(80.5)	3,049	(80.5)
Yes	836	(19.5)	740	(19.5)
Maternal smoking during pregnancy				
No	2,865	(66.9)	2,552	(67.3)
Yes	1,421	(33.2)	1,237	(32.7)
Birth weight (grams)				
< 2500	388	(9.1)	341	(9.0)
≥ 2500	3,891	(90.9)	3,443	(91.0)
Smoking status				
Never smoked	3,441	(82.2)	2,758	(72.9)
Former or current smoker	744	(17.8)	1,028	(27.2)
Weight status				
Normal-weight	3,085	(75.9)	2,015	(56.9)
Overweight	648	(15.9)	953	(26.9)
Obese	332	(8.2)	572	(16.2)
Allergic Rhinitis				
No	3,391	(79.1)	2,858	(75.4)
Yes	895	(20.9)	931	(24.6)
Atopic dermatitis				
No	3,206	(74.8)	3,250	(85.8)
Yes	1,080	(25.2)	539	(14.2)
Wheezing				
No	2,855	(66.6)	2,626	(69.3)
Yes	1,431	(33.4)	1,163	(30.7)

**Figure 1:**
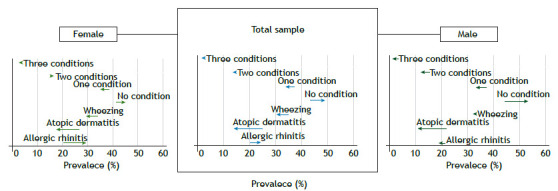
Prevalence (%) of allergic rhinitis, atopic dermatitis, wheezing, and their co-occurrence over follow-up periods in the 1993 Pelotas Birth Cohort, Brazil.

The prevalence of AD, AR, and wheezing, as well as their co-occurrence, are shown in Figure 1. Wheezing was the most reported condition at both follow-ups, with 33.4% (95% CI 32.0 - 34.8) of individuals reporting it at 15 years and 30.7% (95% CI 29.2 - 32.2) at 22 years of age. AR was the least reported health condition at 15 years, while AD held this title at age 22. This pattern persisted when considering sex differences. Regarding the co-occurrence of these three conditions, 43.2% (95% CI 41.7 - 44.7) reported no health condition at 15 years, while almost half the participants (49.3%, 95% CI 47.7 - 50.9) did so at 22 years of age. Approximately one in every six females reported two health conditions at both follow-ups. In contrast, among males, the prevalence suggested a decrease over time (15.0%; 95% IC 13.5 - 16.6 at 15 years vs. 11.8%; 95% CI 10.4 - 13.4 at 22 years). 

**Figure 2:**
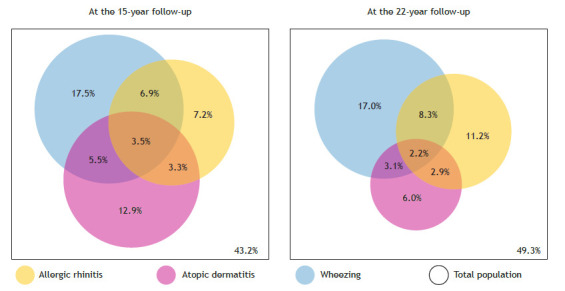
Venn diagram showing the proportions of allergic rhinitis, atopic dermatitis, and wheezing in the 1993 Pelotas Birth Cohort, Brazil.

The intersection between AR, AD, and wheezing is presented in Figure 2. The overlap between AR and wheezing was greater than that of the other conditions, with 6.9% at the 15-year follow-up and 8.3% at the 22-year follow-up. The co-occurrence of all three conditions appeared to decline over time, from 3.5% at 15 years to 2.2% at 22 years.

The co-occurrence of AR, AD, and wheezing, according to demographic, socioeconomic, behavioral, and health characteristics at both follow-ups, is shown in Table 2. At 15 years, White individuals most frequently reported “three conditions” (4.1%; p<0.001), while at 22 years, they mostly reported “two conditions” (15.6%; p<0.001). At 22 years, males predominantly reported “no health condition” (53.6%; p<0.001), whereas females exhibited a higher frequency of “two conditions” (16.4%; p<0.001) and “three conditions” (2.8%; p=0.018).

**Table 2:** Co-occurrence of allergic rhinitis, atopic dermatitis, and wheezing at the 15- and 22-year follow-ups: the 1993 Pelotas Birth Cohort, Brazil.

	At the 15-year follow-up (%)				At the 22-year follow-up (%)			
	No condition	One condition	Two conditions	Three conditions	No condition	One condition	Two conditions	Three conditions
	(n = 1,851)	(n = 1,613)	(n = 673)	(n = 149)	(n = 1,867)	(n = 1,296)	(n = 541)	(n = 85)
Sex								
Male	44.8	36.6	15.0	3.7	53.6	33.0	11.8	1.6
Female	41.7	38.6	16.4	3.3	45.5	35.3	16.4	2.8
p-value	0.044	0.164	0.214	0.483	p<0.001	0.135	p<0.001	0.018
Skin color								
Black/Mixed	43.8	39.8	14.2	2.2	54.3	32.4	11.7	1.6
White	42.9	36.6	16.4	4.1	46.9	35.0	15.6	2.5
p-value	0.587	0.049	0.072	p<0.001	p<0.001	0.128	p<0.001	0.101
Maternal age (years)								
≤ 19	40.2	40.5	16.6	2.9	53.3	32.7	12.5	1.5
20 - 29	43.8	37.1	15.5	3.6	48.6	34.4	14.3	2.7
30 - 39	43.2	37.0	16.2	3.6	48.3	34.2	15.5	1.9
≥ 40	50.0	38.0	8.7	3.3	45.2	41.7	11.9	1.2
p-value	0.189	0.420	0.247	0.786	0.130	0.425	0.326	0.244
Maternal education (years)								
0 - 4	47.7	36.6	12.3	3.4	54.2	34.1	10.2	1.5
5 - 8	43.0	39.1	15.2	2.6	52.4	32.9	12.6	2.1
9 - 11	38.8	36.6	20.0	4.6	37.8	37.8	21.5	2.9
≥12	38.3	34.7	21.3	5.8	41.6	33.4	21.0	3.9
p-value	p<0.001	0.250	p<0.001	p<0.001	p<0.001	0.142	p<0.001	0.044
Income (minimum wage)								
≤ 1	45.8	40.1	12.0	2.2	55.3	33.9	10.0	0.8
1.1 - 3	44.6	36.8	15.8	2.9	50.6	34.1	13.2	2.1
3.1 - 6	39.1	40.4	16.9	3.5	47.4	33.8	15.9	2.9
6.1 - 10	43.5	32.4	18.9	5.1	43.3	36.9	17.0	2.9
> 10	40.0	34.2	17.7	8.1	40.9	32.4	22.8	3.9
p-value	0.021	0.024	0.012	p<0.001	p<0.001	0.830	p<0.001	0.014
Family history of asthma								
No	43.9	36.8	15.9	3.4	49.4	33.7	14.5	2.4
Yes	40.3	41.0	14.7	4.0	48.7	36.4	13.2	1.8
p-value	0.061	0.024	0.381	0.407	0.704	0.170	0.37	0.319
Maternal smoking during pregnancy								
No	43.3	36.7	16.4	3.6	48.8	33.0	15.6	2.7
Yes	43.0	39.6	14.3	3.2	50.3	36.8	11.6	1.4
p-value	0.860	0.068	0.073	0.436	0.387	0.020	p<0.001	0.012
Birth weight (grams)								
< 2500	42.3	41.8	13.4	2.6	48.4	35.8	14.1	1.8
≥ 2500	43.3	37.2	16.0	3.6	49.4	34.0	14.3	2.3
p-value	0.701	0.077	0.187	0.308	0.735	0.519	0.903	0.525
Smoking status								
Never smoked	43.7	37.1	15.9	3.2	51.2	32.5	14.0	2.3
Former or current smoker	40.7	39.9	14.8	4.6	44.1	38.8	15.0	2.1
p-value	0.136	0.156	0.438	0.069	p<0.001	p<0.001	0.441	0.790
Weight status								
Normal-weight	44.6	37.3	15	3.1	50.4	33.2	14.0	2.4
Overweight	38.7	38.9	18.2	4.2	50.6	34.0	13.6	1.8
Obese	36.1	41.0	18.7	4.2	46.0	36.7	14.5	2.8
p-value	p<0.01	0.351	0.045	0.224	0.144	0.294	0.894	0.401

“No health condition” was reported more frequently by individuals with lower maternal education and lower family income at both follow-ups. Approximately one in five individuals with higher maternal education reported having “two conditions” at both follow-ups. At the 22-year follow-up, “one condition” was predominantly reported by individuals whose mothers smoked during pregnancy (36.8%; p=0.020), while “two conditions” (11.6%; p<0.001) and “three conditions” (2.7%; p=0.012) were reported less frequently. More than half of the participants who never smoked reported having “no health condition” at 22 years, compared to those who were former or current smokers (51.2% vs. 44.1%; p<0.001) (Table 2).

The eight potential combinations of AR, AD, and wheezing, along with their respective O/E ratios for the total sample and sex-stratified groups, are shown in Table 3. The frequency of different clusters with one health condition was observed to be lower than expected by chance at both follow-ups. In contrast, the frequency of clusters with two conditions, specifically the combination of AR and wheezing, was found to be higher than expected. This was statistically significant for both males (O/E = 1.4; 95% CI 1.2 - 1.6) and females (O/E = 1.3; 95% CI 1.1 - 1.5) at 15 years, as well as for the total sample at 22 years (O/E = 1.3; 95% CI 1.1 - 1.5). The co-occurrence of all three health conditions was observed to be greater than expected, being nearly double (95% CI 1.2 - 3.4) at 15 years and 2.1 times greater (95% CI 1.4 - 3.0) at 22 years (Table 3).

**Table 3:** Prevalence and co-occurrence of allergic rhinitis, atopic dermatitis, and wheezing at the 15- and 22-year follow-ups: the 1993 Pelotas Birth Cohort, Brazil.

At the 15-year follow-up																			
				Total sample				Female				Male			
No. of cond.	AR	AD	W	O (%)	E (%)	O/E	95% CI	O (%)	E (%)	O/E	95% CI	O (%)	E (%)	O/E	95% CI
0	0	0	0	43.2	39.4	1.1	0.9 - 1.2	41.7	38.4	1.1	1.0 - 1.1	44.8	40.5	1.1	1.1 - 1.2
1	1	0	0	7.2	10.4	0.7	0.5 - 0.9	7.2	9.9	0.7	0.6 - 0.8	7.2	11.0	0.7	0.6 - 0.7
1	0	1	0	12.9	13.3	1.0	0.8 - 1.2	13.7	14.1	1.0	0.9 - 1.1	12.1	12.4	1.0	0.9 - 1.1
1	0	0	1	17.5	19.8	0.9	0.8 - 1.1	17.8	19.8	0.9	0.8 - 1.0	17.3	19.8	0.9	0.8 - 0.9
2	1	1	0	3.3	3.5	0.9	0.6 - 1.5	3.4	3.6	0.9	0.8 - 1.2	3.2	3.4	0.9	0.8 - 1.2
2	0	1	1	5.5	6.7	0.8	0.6 - 1.1	6.4	7.3	0.9	0.8 - 1.0	4.5	6.1	0.7	0.6 - 0.9
2	1	0	1	6.9	5.2	1.3	0.9 - 1.8	6.5	5.1	1.3	1.1 - 1.5	7.3	5.4	1.4	1.2 - 1.6
3	1	1	1	3.5	1.8	2.0	1.1 - 3.4	3.3	1.9	1.8	1.3 - 2.3	3.7	1.7	2.2	1.7 - 2.9
At the 22-year follow-up																			
				Total sample				Female				Male			
No. of cond.	AR	AD	W	O (%)	E (%)	O/E	95% CI	O (%)	E (%)	O/E	95% CI	O (%)	E (%)	O/E	95% CI
0	0	0	0	49.3	44.8	1.1	1.1 - 1.2	45.5	41.0	1.1	1.1 - 1.2	53.6	49.3	1.1	1.0 - 1.1
1	1	0	0	11.2	14.6	0.8	0.7 - 0.9	13.8	17.2	0.8	0.7 - 0.9	8.2	11.5	0.7	0.6 - 0.8
1	0	1	0	6.0	7.4	0.8	0.7 - 1.0	7.2	8.7	0.8	0.7 - 1.0	4.6	5.8	0.8	0.7 - 1-0
1	0	0	1	17.1	19.9	0.9	0.8 - 0.9	14.3	17.1	0.8	0.8 - 0.9	20.1	23.3	0.9	0.8 - 0.9
2	1	1	0	2.9	2.4	1.2	0.9 - 1.6	4.1	3.7	1.1	0.9 - 1.4	1.5	1.4	1.1	0.8 - 1.6
2	0	1	1	3.1	3.3	0.9	0.7 - 1.2	3.4	3.6	0.9	0.7 - 1.2	2.8	2.8	1.0	0.8 - 1.3
2	1	0	1	8.3	6.5	1.3	1.1 - 1.5	8.9	7.2	1.2	1.1 - 1.5	7.6	5.4	1.4	1.2 - 1.7
3	1	1	1	2.2	1.1	2.1	1.4 - 3.0	2.8	1.5	1.8	1.3 - 2.5	1.6	0.6	2.5	1.6 - 4.1

No. of cond.: number of conditions; AD: atopic dermatitis; AR: allergic rhinitis; W: wheezing; O: observed value; E: expected value; O/E: observed/expected value; 95% CI: 95% confidence interval.

## DISCUSSION

In this study, wheezing was the most commonly reported health condition at both follow-ups (17.5% at 15 years and 17.0% at 22 years). The co-occurrence of wheezing, AR, and AD appears to have declined over time, from 3.5% at 15 years to 2.2% at 22 years. However, the frequency of this cluster was twice and more than twice as high as expected by chance at the 15-year and 22-year follow-ups, respectively.

A recent systematic review of population-based studies of individuals aged 5-69 years^32^ concluded that the global prevalence of current wheezing was 11.5% (95% CI 9.1 - 14.3). Conversely, the prevalence found in this study was more similar to that reported for ever wheezing (17.9%; 95% CI 14.2 - 22.3). Incorrect diagnoses and poor symptom control increase the risk of asthma exacerbations, which can manifest as wheezing and dyspnea. Consequently, due to limited access and/or availability of medication in lower- and upper-middle-income countries, asthma exacerbations may be more common in these regions.^1^ ^,^ ^32^

The global prevalence of AR in adults ranges from 1.0% to 63.0%, depending on geographic location.^6^ In a city in the state of Rio Grande do Sul, Brazil, a ten percentage point decrease in AR prevalence was reported between 2011 and 2018 (63.3% vs. 50.5%).^20^ In this study, the prevalence of AR at both follow-ups was lower than those reported in other studies, with AR being the least reported health condition at 15 years (20.9%), increasing to 24.6% at 22 years. This may be attributed to the prevalence being estimated exclusively based on self-reported medical diagnoses, which can be less accurate in lower- and upper-middle-income countries. In such settings, challenges such as limited access to healthcare, underdiagnosis, and variations in diagnostic criteria often affect the accuracy of these estimates.^33^

Globally, 11.0% of adolescents have experienced AD at some point in their lives.^4^ Among adults, the prevalence of AD varies widely, ranging from 3.4% in Israel to 33.7% in Thailand.^5^ In Brazil, the overall prevalence is around 9.2%, with the highest estimate found in the Southeast region and lower prevalence among individuals aged 18-24 years.^5^ In the present study, the frequency of AD was higher when compared to the national average but decreased substantially from 25.2% at age 15 to 14.2% at age 22. Since this health condition is often confused with other skin disorders,^4^ ^,^ ^5^ ^,^ ^7^ misdiagnosis or incorrect diagnosis can lead to inflated prevalence rates, which may help explain the decreased prevalence observed at age 22.^33^

The high prevalence of allergic asthma is thought to be attributed to shared genetic predispositions and common environmental exposures.^14^ ^,^ ^16^ ^,^ ^17^ In this study, however, wheezing was most frequently reported as an isolated condition rather than co-occurring with other atopic diseases. This may be due to the fact that asthma can be non-allergic, cough-variant, adult-onset, associated with persistent airflow limitation, exercise-induced, or linked to obesity.^1^ ^,^ ^34^ Additionally, since wheezing was analyzed as a proxy for asthma rather than through self-reported medical diagnosis, like AR and AD, there is a possibility of misdiagnosis or incorrect diagnosis.

Only 3.5% of the participants at age 15 and 2.2% at age 22 reported the co-occurrence of AR, AD, and wheezing. Similar results were observed by Pedersen et al. (2020) in a rural population in India.^16^ Although both studies differ in their geographic and socioeconomic contexts, their findings indicate that, despite the likely absence of official medical diagnoses, the co-occurrence of these health conditions was more frequent than expected. Thus, both studies suggest that integrated approaches to identifying and managing these three health conditions could be beneficial across various global settings.^16^

Despite the low prevalence of co-occurrence compared to isolated conditions, it was observed more frequently than expected at both follow-ups, with the highest O/E ratio relative to other clusters. This suggests that evaluations should focus on multiple conditions, as this approach can improve patient care and support more effective public health interventions.^10^ ^,^ ^16^ Recognizing the individual characteristics of each patient and considering all aspects of their health - rather than focusing solely on individual systems - enables the identification of potential interactions between comorbidities.^12^ This holistic perspective not only facilitates improved individual health outcomes but also promotes the efficient allocation of healthcare resources, ultimately leading to a more effective response to the complex interplay of health conditions.^10^ ^,^ ^12^

The presence of one atopic condition significantly increases the likelihood of another.^12^ ^,^ ^16^ ^,^ ^17^ In this study, the co-occurrence of AR and wheezing was found to be more frequent than other combinations and appeared to increase over time. A cross-sectional study showed a strong association between asthma and AR (OR 8.39; 95% CI 6.48 - 10.86).^16^ Other studies suggest that up to 50% of AR cases may present with asthma symptoms, while nasal symptoms are observed in up to 85% of people with asthma.^20^ ^,^ ^35^ Furthermore, having AD is also associated with asthma (OR 5.56; 95% CI 4.26 - 7.26).^16^

Our findings also indicate that the number of conditions reported by participants is strongly associated with sociodemographic factors. Specifically, males were more likely to report having no health conditions compared to females, a trend that was more pronounced at the 22-year follow-up. While AR, AD, and asthma often have higher prevalence rates in males during childhood, this trend can reverse in adulthood.^1^ ^,^ ^20^ In addition, females generally exhibit greater health-seeking behaviors and are more likely to seek medical care for symptoms or health concerns.^36^

Participants from lower socioeconomic backgrounds (e.g., lower maternal education and family income) and Black/Mixed individuals were more likely to report having “no health conditions”. Brazil, known for its significant socioeconomic and ethnic disparities since colonial times, is one of the most unequal countries in the world.^37^ This may explain why White individuals and those in better socioeconomic circumstances often have better access to healthcare and more accurate diagnoses, resulting in the reporting of more health conditions in this study. This highlights an important social gradient in this context.^33^ ^,^ ^37^ ^,^ ^38^ Furthermore, it emphasizes the need for health policies that address these inequalities and promote equitable access to healthcare services for all population groups.^33^

This study had some limitations. First, as a cross-sectional study, it cannot establish a clear temporal association between exposures and outcomes. Second, differences between respondents and non-respondents at both follow-ups may affect the results, particularly due to the social gradient.^33^ Males, as well as participants with low maternal education (0-4 years) and low birth weight, may be underrepresented in the study sample. This nonresponse bias could lead to an underestimation of the co-occurrence of AR, AD, and wheezing in these groups, as their lower use of healthcare services may result in fewer diagnoses being recorded. Third, the variables were measured through self-reporting, which can introduce social desirability and recall biases.^39^

Additionally, observing wheezing alone may not fully capture asthma cases, potentially leading to an underestimation of prevalence. However, according to Sistek et al. (2001), wheezing is the most sensitive single symptom (75.0%) for diagnosing asthma and is considered a proxy for the disease.^40^ On the other hand, there were notable strengths to this study. It utilized data from a cohort of live births, which is representative of the urban area of Pelotas, and achieved high response rates, allowing for the extrapolation of results to similar settings/middle-income countries. Moreover, the training of interviewers to assess reliability ensured the quality of the collected information.

This study highlights the social gradient in the diagnosis and reporting of the co-occurrence of AR, AD, and wheezing. These findings are important for formulating more effective health policies regarding the diagnosis and treatment of these conditions, as well as for allocating targeted resources to the groups most in need of intervention. Additionally, since atopy increases the risk of allergic asthma, individuals with AR and/or AD should be monitored for respiratory symptoms. Further research is needed to address these disparities and improve the early detection and management of AR, AD, and wheezing.
